# Turner’s syndrome associated with discoid lateral meniscus and Blount’s disease: a case report

**DOI:** 10.1186/s12891-021-04336-z

**Published:** 2021-05-15

**Authors:** Tsunemasa Kita, Takuya Tajima, Etsuo Chosa

**Affiliations:** Department of Orthopedic Surgery, Faculty of Medicine, University of Miyazaki, 5200 Kihara, Kiyotake, Miyazaki, 889-1692 Japan

**Keywords:** Turner’s syndrome, Discoid meniscus, Blount’s disease, Case report

## Abstract

**Background:**

Turner’s syndrome, discoid meniscus, and Blount’s disease have all been studied in isolation, but, to the best of our knowledge, there have been no studies reporting a patient with all three. Thus, the first case of Turner’s syndrome with discoid meniscus and Blount’s disease is presented.

**Case presentation:**

A 5-year-old Japanese girl with a history of Turner’s syndrome and Blount’s disease complained of pain in her left knee. Magnetic resonance imaging showed a discoid lateral meniscus tear, and arthroscopic partial meniscectomy was performed, providing a good outcome.

**Conclusions:**

In this report, some possible explanations regarding the concomitant presence of these three diseases are discussed. A possible explanation in this case is that the patient with Turner’s syndrome had a discoid lateral meniscus that might have been induced by some genetic factors associated with Turner’s syndrome, and then the discoid lateral meniscus might have been the mechanical stress that caused Blount’s disease.

## Background

Turner’s syndrome results from the complete or partial loss of an X chromosome in females, and it affects about 1 in 2500 live-born females [1]. Turner’s syndrome is mainly characterized by short stature, ovarian failure, congenital malformations of the heart, endocrine disorders, osteoporosis, and autoimmune disorders [2].

Discoid meniscus is a diagnosis that encompasses a spectrum of meniscal disorders of shape and stability [3]. There is a higher prevalence of discoid meniscus in Asian countries than in Western countries: 13% in Japan, 10.6% in Korea, 5.8% in India, and 3 to 5% in the United States [3, 4]. It has been suggested that discoid meniscus is a congenital disease, and genetic or familial factors may account for some of the causes [3–5].

Blount’s disease is a pathologic varus deformity that results from disruption of normal cartilage growth at the medial aspect of the proximal tibial physis [6, 7]. It may cause severe varus deformity, leg length discrepancy, and articular incongruity [7]. It has been suggested that risk factors for this disease include early ambulation, obesity, and African or Scandinavian descent, but the exact etiology and pathophysiology remain unknown [6–8].

Turner’s syndrome, discoid meniscus, and Blount’s disease have all been studied in isolation, but, to the best of our knowledge, there have been no reports of patients with all three conditions.

The case of a patient with Turner’s syndrome and Blount’s disease who underwent arthroscopic partial meniscectomy for a discoid lateral meniscus tear is presented.

## Case presentation

A 5-year-old Japanese girl had a 6-month history of pain in her left knee while walking. She then developed increased pain, and knee extension was restricted, with no history of trauma.

**Fig. 1:**
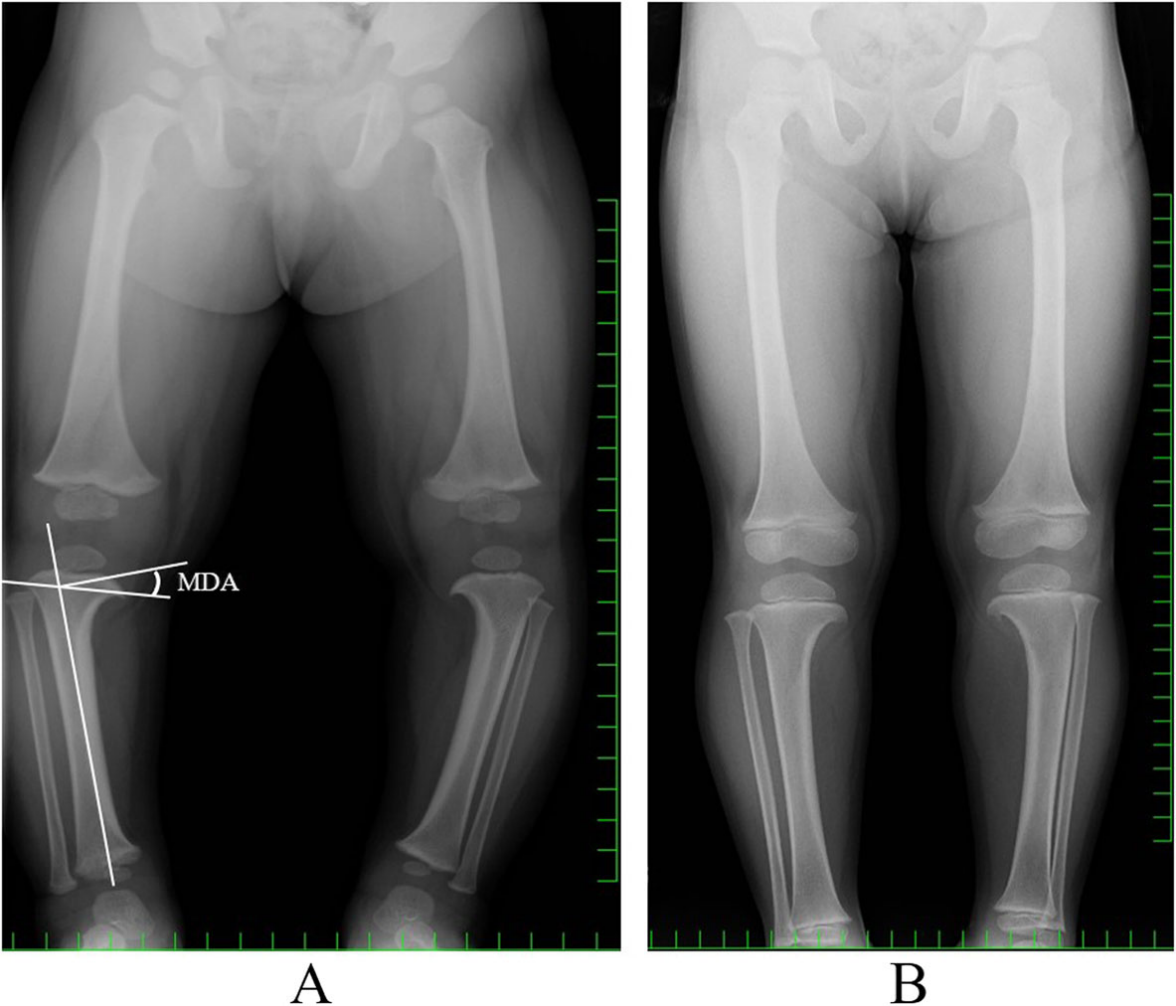
**a** Anteroposterior radiograph of the legs at 1 year and 3 months when the patient was diagnosed with Blount’s disease. Medial beaking and downward slope of the proximal tibia are seen. The metaphyseal-diaphyseal angle (MDA) is 20° on the right and 23° on the left. **b** Anteroposterior radiograph of the legs at the age of 3 years. Medial beaking and downward slope of the proximal tibia are not noticeable. The MDA is 10° on the right and 11° on the left

**Fig. 2:**
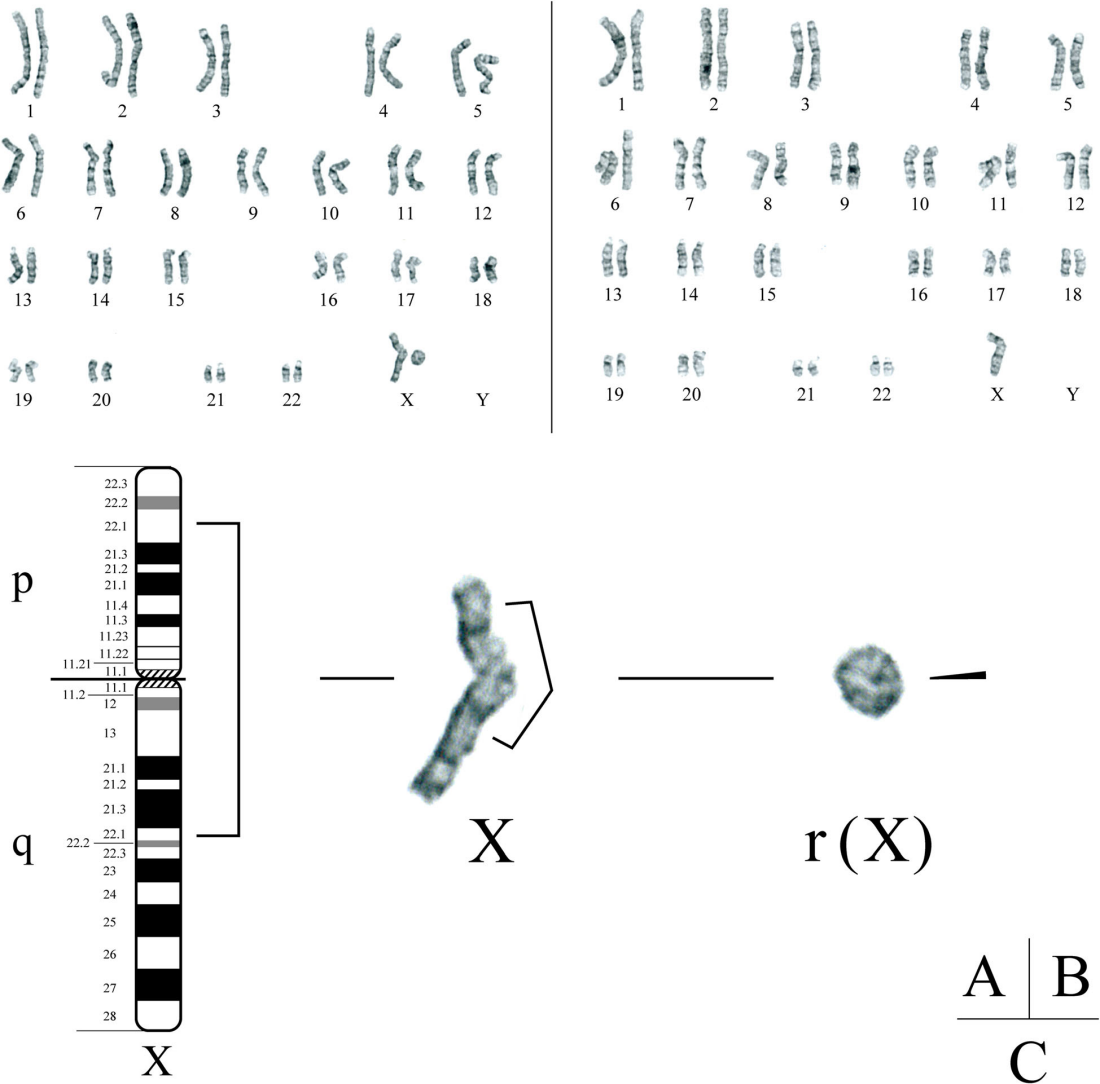
Chromosomal analysis of this patient showing a mosaic pattern. **a** Karyotype of 46, X, r (X) (p22.1 q22.1). **b** Karyotype of 45, X. **c** The detailed analysis of (A) shows partial monosomy with deletion of p22.1-pter and q22.1-qter

She had been diagnosed with Blount’s disease on radiography at our institute at the age of 1 year and 3 months. On radiographs at that time, varus deformity of the proximal tibia with medial beaking and a downward slope of the proximal tibia (Grade 1 in the Langenskiold classification) were confirmed. The metaphyseal-diaphyseal angle (MDA) on radiography was 20° on the right and 23° on the left (Fig. 1 a). She had been using a knee brace up to the age of 3 years, with a significant improvement; the MDA was 10° on the right and 11° on the left (Fig. 1 b). In addition, she had also been diagnosed with Turner’s syndrome at the age of 2 years and 6 months by chromosomal analysis that showed the karyotype of 46, X, r (X) (p22.1 q22.1)/45, X (Fig. 2). When she was diagnosed, her height and weight were 80.0 cm (− 2.4 SD) and 9.8 kg (− 2.0 SD), respectively. She was then started on growth hormone injections (0.35 mg/kg/week). Besides short stature, cubitus valgus was found to be associated with Turner’s syndrome. Her family members had never been diagnosed with Turner’s syndrome, discoid meniscus, or Blount’s disease.

**Fig. 3:**
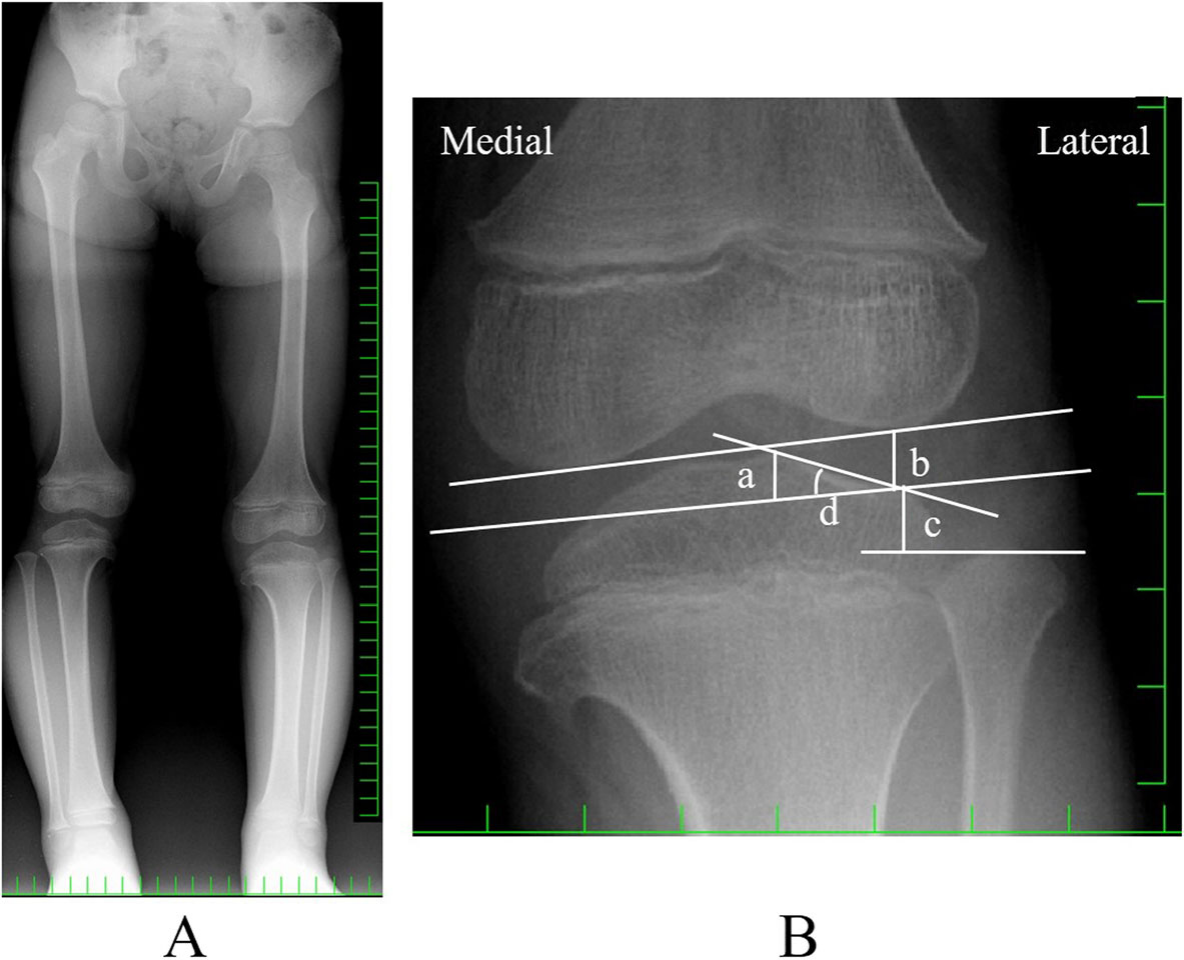
**a** Anteroposterior radiograph of the legs at the age of 5 years. The MDA is 8° on the left and 9° on the right. Medial beaking and downward slope of the proximal tibia have improved. **b** The evaluation for discoid lateral meniscus on anteroposterior radiography. a: Height of the lateral tibial spine, distance from the imaginary tibial joint line to the tip of the lateral intercondylar spine. b: Lateral joint space distance, from the imaginary tibial joint line to the lateral femoral condylar joint line at its midportion. **c** Height of the fibular head, from the imaginary tibial joint line to the tip of the fibular head. **d** Obliquity of the lateral tibial plateau angle, formed by the imaginary tibial joint line and the articular line of the lateral tibial plateau

**Fig. 4:**
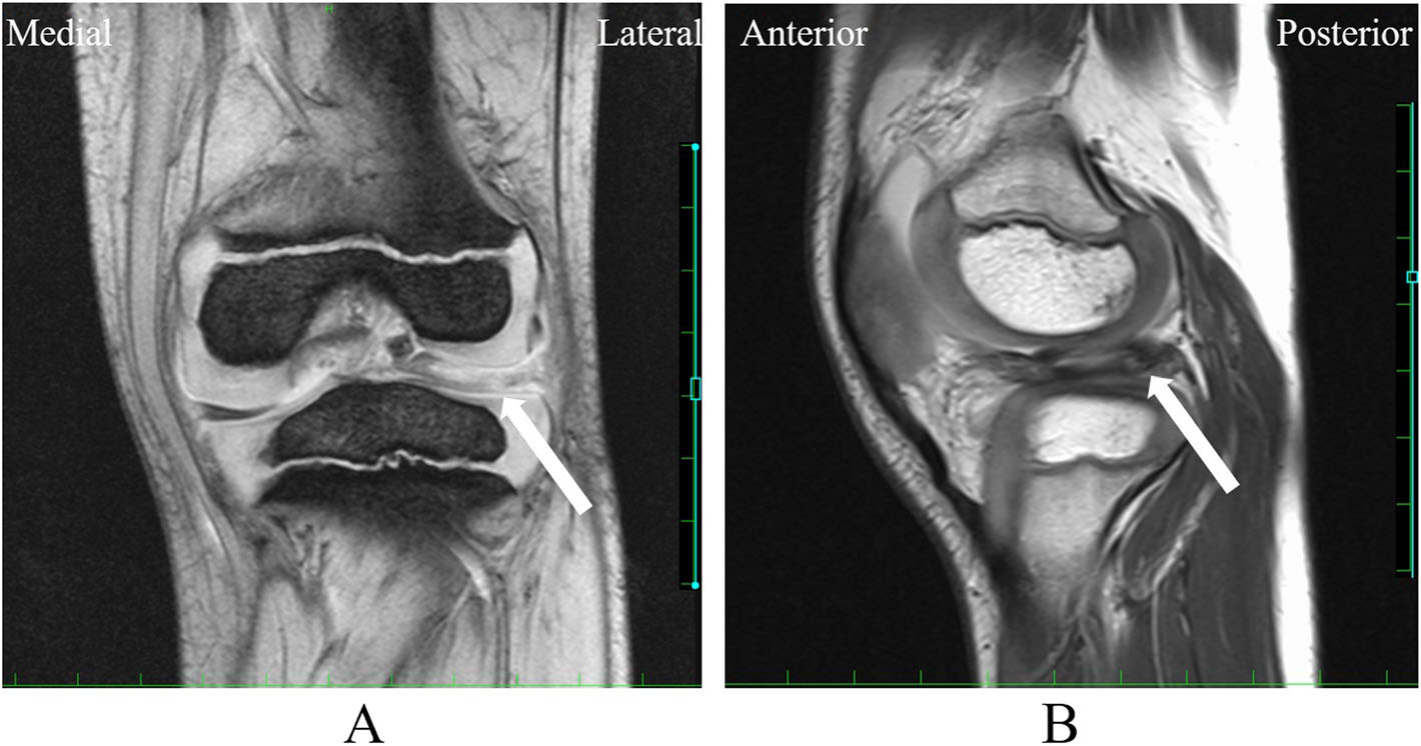
Magnetic resonance imaging (MRI) coronal (**a**) and sagittal (**b**) views of the left knee. There is a thickened lateral meniscus, suggesting a discoid meniscus (Arrow)

At the age of 5 years, when she complained of increased knee pain, the physical findings were as follows. Height and weight were 98.7 cm (− 2.0 SD) and 15 kg (− 1.0 SD), respectively. Both tibias showed slight bowing. She walked with a limp, exhibiting motion pain and tenderness at the medial/lateral joint space of the left knee. Extension of the left knee was restricted, with a range of motion of − 15° to 140°. McMurray’s test was positive with internal/external rotation. There were no symptoms or signs related to the right side. On radiography, the MDA was 8° on the right and 9° on the left. Medial beaking and downward sloping of the proximal tibia were not noticeable (Fig. 3 a). To diagnose discoid lateral meniscus, the height of the lateral tibial spine, lateral joint space distance, height of the fibular head, and obliquity of the lateral tibial plateau were measured, according to Choi’s method [9] (Fig. 3 b) (Table 1). Her left knee showed tibial eminence hypoplasia, fibular head elevation, and greater obliquity of the lateral tibial plateau, which suggested discoid lateral meniscus. Magnetic resonance imaging (MRI) revealed a thickened lateral meniscus that almost completely surrounded the tibial lateral plateau. Although the ratio of minimal meniscal width to maximal tibial width was difficult to measure, it was clearly greater than 20%. The lateral meniscus was severely damaged and torn, which caused signal variation of the meniscus. It was suspected that the meniscal fragment was locking into the lateral joint. There was a large effusion and synovial proliferation. There were no particular changes in other components, such as the medial meniscus, anterior cruciate ligament (ACL), posterior cruciate ligament (PCL), articular cartilage, and so on. There was no space-occupying lesion on MRI (Fig. 4 a,b).

**Table 1:** Parameters for radiographic evaluation of discoid lateral meniscus

Parameter	Measured value Right/Left	Cut off value
Height of the lateral tibial spine (a)	2.8/5.0 mm	< 6 mm
Lateral joint space distance (b)	6.6/6.4 mm	> 8 mm
Height of the fibular head (c)	16.0/6.6 mm	< 14.9 mm
Obliquity of the lateral tibial plateau (d)	26.9/24.4°	> 17.6°

**Fig. 5:**
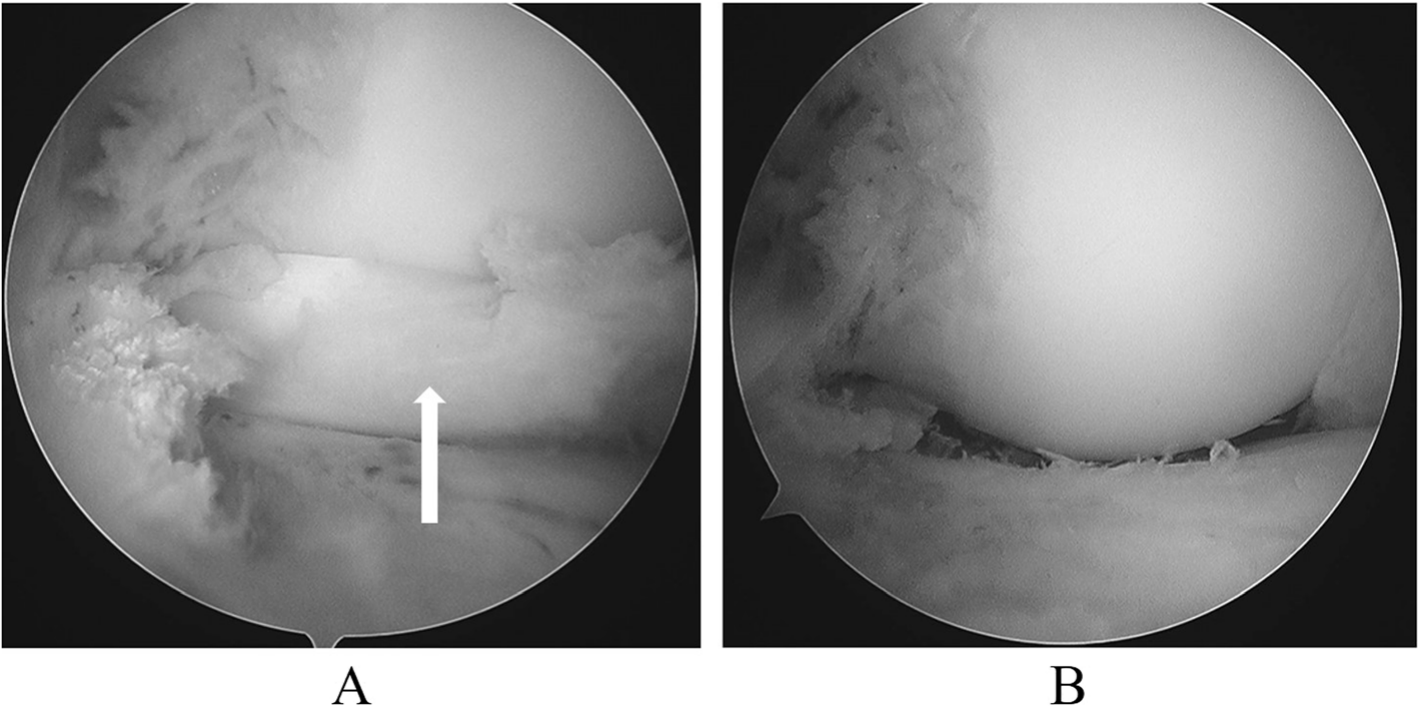
Arthroscopic findings. **a** The discoid lateral meniscus appears to be locking in the lateral joint (Arrow). **b** The discoid lateral meniscus is largely alleviated

An arthroscopic procedure was performed. First, at the patello-femoral joint, plica was found, Sakakibara type B. Medial meniscus, ACL, and PCL injuries were not seen. Subsequently, a thickened discoid lateral meniscus almost completely covering the lateral tibial plateau was found. The lateral meniscus was completely torn, and the fragment was locking into the lateral joint space (Fig. 5a). It clearly restricted knee extension. There was no noticeable damage of the articular cartilage. Using a clamp and a radio-frequency device, the inner region including the injured meniscus was elevated, and a crescent shape was formed, resulting in good stability and shape (Fig. 5b). The restriction of knee extension was improved by the procedure.

Postoperative rehabilitation consisted of range of motion exercises, muscle-strengthening exercises, and walking exercises. The patient’s pain and restricted knee extension were finally relieved, and she started playing table tennis in junior high school. The symptoms of Blount’s disease have not relapsed; the MDA at the age of 15 years was 8° for both knees. For Turner’s syndrome, she continues to receive growth hormone treatment.

## Discussion and conclusions

Turner’s syndrome is known to cause morphological defects of the skeletal system, including abnormal knee alignment. However, combinations of Turner’s syndrome with discoid meniscus and Turner’s syndrome with Blount’s disease have never been reported. In this report, each of these relationships is considered.

### Turner’s syndrome with discoid meniscus

A particular gene mutation may be the cause of the occurrence of these two diseases. Although it is not the cause of Turner’s syndrome, mutation of the short stature homeobox-containing (SHOX) gene on the X-chromosome is associated with the short stature found in Turner’s syndrome [10]. The estimated prevalence of the SHOX mutation is almost 100% in Turner’s syndrome [11]. SHOX is necessary for the correct growth and maturation of bone. SHOX expression regulates cell proliferation and induces apoptosis in growth plates. SHOX mutation facilitates an abnormal balance between proliferation and subsequent differentiation of the chondrocytes in the growth plate, leading to defective linear growth [12]. In addition, SHOX cooperates with transcription factors, SRY-related high mobility group box 5/6 and 9 (SOX5/SOX6 and SOX9). SOX5/6 and SOX9 activate the Agc1 enhancer that regulates the expression of aggrecan, which is an essential extracellular matrix component of cartilage, and SHOX mutation affects this activation [13–15].

Transforming growth factor-β (TGF-β) is also related to chondrogenesis [15–18]. The TGF-β signal promotes early chondrogenesis through the activation of SOX9, whereas late differentiation and maturation of chondrocytes are inhibited by TGF-β [15–18]. Some studies reported that TGF-β levels were higher in Turner’s syndrome than in cases without Turner’s syndrome [19, 20]. This increase of TGF-β might cause greater inhibition of chondrocyte maturation and a decrease in proper chondrogenesis.

There have been some reports indicating collagen abnormalities in areas other than the growth plate in Turner’s syndrome. Some Turner’s syndrome patients with aortic dissection showed a skewed distribution between collagen types and cystic medial necrosis, which was caused by a background of disrupted elastin fibers with accumulation of excessive amounts of glycosaminoglycans [21, 22]. A study of aborted fetuses with Turner’s syndrome showed abnormal extracellular matrix of the nuchal skin, with increased amounts of glycosaminoglycans [23]. If such a collagen abnormality is also present in the meniscus, collagen-related factors including SHOX or TGF-β might contribute to promoting discoid meniscus.

### Turner’s syndrome with Blount’s disease

Patients with Turner’s syndrome sometimes have abnormal knee alignment, primarily genu valgum [1, 2, 10]. Although it is rare, Turner’s syndrome causes genu varus of the proximal tibia because of premature epiphyseal closure in the medial proximal tibia [24]. As the mechanism of development, genu varus in Turner’s syndrome and Blount’s disease may be the same in terms of the failure of normal cartilage growth in the medial proximal tibia. Some factors in Turner’s syndrome might affect the occurrence of Blount’s disease, although it is unlikely, because Turner’s syndrome usually presents with genu valgum.

### Blount’s disease with discoid meniscus

It has been reported that children with Blount’s disease have increased thickness of the unossified cartilage of the proximal medial tibial epiphysis, with increased height and width of the medial meniscus [25]. In contrast, the morphologies of the unossified cartilage and the meniscus in the lateral compartment did not differ from those of normal patients [25]. In this regard, Blount’s disease is less likely to be the cause of discoid lateral meniscus.

As mentioned above, genetic factors and mechanical overloads such as early ambulation and obesity are suggested to be the important causes of Blount’s disease [6, 7, 26]. In the present case, the patient was thin (− 1.0 SD) and had not starting walking early (1 year and 6 months). Although discoid lateral meniscus has never been reported as the cause of Blount’s disease, if it were one of the mechanical stresses on the proximal medial tibial epiphysis, it might affect the occurrence of Blount’s disease. However, not all patients with discoid lateral meniscus have Blount’s disease. Thus, it might be just one of the possible factors promoting Blount’s disease. There may also be other significant causes.

In summary, the etiology of the present case may be explained as follows. The patient with Turner’s syndrome had discoid lateral meniscus that might have been induced by some genetic factors associated with Turner’s syndrome, whereas the discoid lateral meniscus might have been the mechanical stress that caused Blount’s disease. The discoid lateral meniscus was torn because of its vulnerability, but the arthroscopic procedure and postoperative rehabilitation allowed the patient to participate in sports. Blount’s disease has not recurred, and she continues her treatment for Turner’s syndrome. To the best of our knowledge, this is the first report of a patient with all three conditions, Turner’s syndrome, discoid lateral meniscus, and Blount’s disease.

## Data Availability

Data sharing is not applicable to this article as no datasets were generated or analyzed during the current study.

## Abbreviations

MDA: Metaphyseal-diaphyseal angle
SHOX gene: Short stature homeobox-containing gene
SOX: SRY-related high mobility group box
TGF-β: Transforming growth factor-β
